# Improving Digital Cancer Care for Older Black Adults: Qualitative Study

**DOI:** 10.2196/63324

**Published:** 2025-02-19

**Authors:** Paul Wankah, Shivani Chandra, Aisha Lofters, Nebila Mohamednur, Beverley Osei, Tutsirai Makuwaza, Ambreen Sayani

**Affiliations:** 1 Faculty of Dental Medicine and Oral Health Sciences McGill University Montreal, QC Canada; 2 Women's College Hospital Toronto, ON Canada; 3 University of Toronto Toronto, ON Canada

**Keywords:** digital care, cancer care, older Black adults, health equity, social determinants of health, access to care, health quality

## Abstract

**Background:**

Health systems are rapidly promoting digital cancer care models to improve cancer care of their populations. However, there is growing evidence that digital cancer care can exacerbate inequities in cancer care for communities experiencing social disadvantage, such as Black communities. Despite the increasing recognition that older Black adults face significant challenges in accessing and using health care services due to multiple socioeconomic and systemic factors, there is still limited evidence regarding how older Black adults’ access and use digital cancer care.

**Objective:**

This study aims to better understand the digital cancer care experience of older Black adults, their caregivers, and health care providers to identify strategies that can better support patient-centered digital cancer care.

**Methods:**

A total of 6 focus group interviews were conducted with older Black adults living with cancer, caregivers, and health care providers (N=55 participants) across 10 Canadian provinces. Focus group interviews were recorded and transcribed. Through a theory-informed thematic analysis approach, experienced qualitative researchers used the Patient Centered Care model and the synergies of oppression conceptual lens to inductively and deductively code interview transcripts in order to develop key themes that captured the digital cancer care experiences of older Black adults.

**Results:**

In total, 5 overarching themes describe the experience of older Black adults, caregivers, and health care providers in accessing and using digital cancer care: (1) barriers to access and participation in digital care services, (2) shifting caregivers’ dynamics, (3) autonomy of choice and choosing based on the purpose of care, (4) digital accessibility, and (5) effective digital communication. We identify 8 barriers and 6 facilitators to optimal digital cancer for older Black adults. Barriers include limited digital literacy, linguistic barriers in traditional African or Caribbean languages, and patient concerns of shifting power dynamics when supported by their children for digital cancer care; and facilitators include community-based cancer support groups, caregiver support, and key features of digital technologies.

**Conclusions:**

These findings revealed a multifaceted range of barriers and facilitators to digital cancer care for older Black adults. This means that a multipronged approach that simultaneously focuses on addressing barriers and leveraging community strengths can improve access and usage of digital cancer care. A redesign of digital cancer care programs, tailored to the needs of most structurally marginalized groups like older Black adults, can enhance the digital care experience for all population groups. Public policies and organizational practices that address issues like availability of internet in remote areas, resources to support linguistic barriers, or culturally sensitive training are important in responding to the complexity of access to digital l cancer care. These findings have implications for other structurally marginalized and underresourced communities that have suboptimal access and usage of digital care.

## Introduction

Digital care is defined as “the secure use of information and communication technologies in support of health and health-related fields, including health care services, health surveillance, health literature, and health education, knowledge, and research” [1]. Used in complement with or in lieu of in-person encounters, digital care encompasses a wide array of modalities, including phone calls, videoconferencing, remote monitoring, asynchronous messaging (eg, email and texting), and the use of a patient portal [1]. Digital cancer care has been shown to be effective for pretreatment discussions, the monitoring of adverse events, for counselling purposes, and for long-term cancer care follow-up [2]. However, there is a growing body of evidence that demonstrates inequitable access to digital care services, resulting in a widening health gap between population groups that are able to use digital care versus those that cannot. Reasons for this include an inability to afford the necessary technology, lack of language accessibility within certain applications, lack of infrastructure to support digital connectivity in rural areas, lack of cultural relevance of care provision, and lower levels of comfort in using certain technologies [3-6].

Structurally underresourced [7] and marginalized population subgroups, such as older Black adults, experience barriers specific to their identity, such as physical, cognitive, and sensory limitations or concerns [8]; lack of social or peer support to help with the use of technology [9]; and preference for in-person visits due to limited trust in technology and concerns about privacy [10]. When services are available, acceptability of care [11] is determined by a lack of cultural tailoring of language used in service and technology, such as not adapting the linguistic content of eHealth platforms to the cultural preferences of diverse populations [12], varying views or beliefs for disease management, such as cultural barriers when shifting from traditional in-person care to technology-based intervention platforms [13], and existing historic and societal barriers, such as general distrust of health care providers [14].

While other countries report that cancer disproportionately affects Black people [15], the lack of comprehensive race-based data collection in Canada means that the extent of the care gap affecting Black Canadians is unknown. However, a recent study that used census data to explore patterns of mortality inequities of Black adults in Canada indicates that Black men had an increased risk of dying from prostate cancer (hazard ratio: 1.33) than their White counterparts, and Black women were at an increased risk of dying from stomach cancer (hazard ratio 1.76), corpus uteri cancer (hazard ratio 1.78), and lymphomas and multiple myelomas (hazard ratio 1.26) than their White counterparts [16]. With limited race-based data collection, it is also unclear how race and racism affect digital cancer care use in Canada. It is essential that digital cancer care is delivered in a manner that meets the needs of all communities and does not exacerbate inequities between the most privileged and the most underresourced communities. Knowledge generated in partnership with these communities is essential to developing health care policy and planning that focuses on enhancing equity in access to and quality of digital care. Hence, the purpose of this study was to better understand the digital cancer care experiences of older Black cancer patients, their caregivers, and health care providers. Through this understanding, we sought to identify strategies that can support the delivery of better patient-centered digital cancer care.

## Methods

### Study Design

Qualitative research methods are appropriate for exploring the lived experiences and perspectives of individuals who are exposed to a social phenomenon like the usage of digital cancer care [17,18]. We used theoretical thematic analysis [17] as a methodology in order to conceptualize, collect, organize, and interpret data for this study. Specifically, we used a codebook approach to thematic analysis [18] that combines structure and reflexivity in methodological choices. In the following subsections of the methods, we describe how we structured our study approach by using 2 theoretical frameworks—the synergies of oppression lens [19] and the patient-centered care model [20]—to create a codebook and using multiple coders to ensure the accuracy of the coding process. The research team’s interest in this study stems from a commitment to addressing systemic inequities and amplifying the voices of Black communities. We recognize our positionality influences every stage of the research process, from framing the research question to interpreting findings. Researcher subjectivity was addressed by regular team meetings to discuss and interpret data.

#### Theoretical Approach

The synergies of the oppression lens [19] is an analytic lens that can be used to illuminate the intersection of multiple systems of oppression that shape the lived or living experiences of older Black patients with cancer as they navigate the health system. Characteristics such as race, age, level of disability, sex at birth and gender, and income levels of individual and social groups are powerful indicators of their access to material and social resources that are necessary to achieve health and wellness. These characteristics (or multiple oppressions) operate in complex synergistic patterns that lead to poor health and have been used previously to unpack and understand inequities in cancer care [21-25]. In this study, we have used the synergies of oppression analytical lens to theorize a combination of oppressions impacting the digital care experiences of older Black patients with cancer. We were also guided by the 7 dimensions of Patient-Centred Care [20] model that outlines the essential components of a patient-responsive health care as (1) aligning the health system’s vision, mission, values and quality improvement drivers to patient-centered goals; (2) providing collaborative, coordinated and accessible care at the right time and the right place; (3) providing care that considers physical comfort and emotional well-being; (4) respecting patient and family preferences, values, and cultural traditions in the organization and delivery of care; (5) integrating family and patients in care teams, and having them fully partake in decision-making at the clinical and system level; (6) encouraging and facilitating the presence of family members in care settings; and (7) supporting informed decision-making by patients and their family by providing complete information in a timely manner. Accordingly, our study questions were as follows: What is the experience of older Black patients who have used digital cancer care? How can we improve the digital cancer care experience of older Black patients with cancer?

We hypothesize that strategies and interventions that will improve the digital cancer care experience of populations that are experiencing the most structural marginalization will improve the digital cancer care experience across all population groups in alignment with Bell Hooks’s concept of “centering the margins” [26,27].

#### Participant Recruitment and Setting

This study was carried out between May 2022 and March 2023. A total of 3 groups of study participants were the focus of this study (Textbox 1).

**Three groups of study participants.**

1. Patients: comprised of older Black adults, aged 55 years or older, given the growing health and social challenges of this age group, which includes chronic disease management and barriers in access to high-quality and timely care [28], who had been diagnosed with any form of cancer, including those no longer in active treatment, and had used digital cancer care services across the 10 Canadian provinces.
2. Caregivers: comprised of any individual who provided informal support in activities of daily living to older Black patients with cancer—this included family or paid caregivers.
3. Providers: comprised of health care professionals who regularly delivered clinical care to older Black patients with cancer.

Sampling was carried out through a nonprobabilistic convenience approach. This sampling strategy allowed us to cover various contexts (urban, rural, and remote) and diverse informants who could provide rich data on their experiences in receiving and delivering digital cancer care to older Black patients. The recruitment of potential study participants was supported by the Canadian Cancer Society (CCS), which is the largest cancer charity in Canada. Potential participant recruitment started in August 2022 and ended in January 2023. The CCS posted invitations to participate in the study using their database of patients with cancer, caregivers, and health care professionals. Those who were interested in participating in the study contacted the project coordinator by email—the research team received about 600 responses from potential participants. We sent detailed information letters on the study to all potential participants. In total, 6 groups of participants were created for each focus group, considering time zones and provincial linguistic diversity: (1) patients and caregivers in the Eastern provinces (Newfoundland and Labrador, Nova Scotia, and Prince Edward Island); (2) patients and caregivers in Central or Prairies provinces (Ontario and Manitoba); (3) patients and caregivers in Western or Prairies provinces (Saskatchewan, Alberta, and British Columbia); (4) patients and caregivers in Francophone/Bilingual provinces (Quebec and New Brunswick); (5) health care providers in 8 Canadian provinces; and (6) health care providers in Francophone or bilingual provinces (Quebec and New Brunswick); see Table 1. The final study comprised 55 participants across the 6 groups (Table 1). The final sample of 55 participants was sufficient to achieve code saturation in the qualitative data analysis, ensuring no new themes or codes emerged from the data [29,30].

**Table 1:** Number of participants in the focus groups.

Focus group		Participants, n
**Patients with lived experience and caregivers**		
	Eastern Provinces (NF, NB, NS, and PEI)	12
	Central & Prairie Provinces (ON and MB)	10
	Western & Prairie Provinces (BC, AB, and SK)	10
	Francophone or bilingual provinces (QC and NB)	8
**Health care providers**		
	English-speaking provinces	10
	Francophone or bilingual provinces	5
Total		55

### Data Collection

Data were collected through 6 focus group interviews (about 120 minutes duration for each focus group) with the aforementioned groups of participants (Table 1). Focus group interviews were led by 2 Black peer researchers (PW and TM). This was an intentional and important strategy to reduce mistrust of the research process and to increase study participation by leveraging existing relationships between Black communities in Canada. During the focus group interviews, we used 2 distinct interview guides—a patient or caregiver interview guide (Multimedia Appendix 1) and a health care provider interview guide (Multimedia Appendix 2)—to guide data collection. Both interview guides were developed from the research objectives and targeted literature review. Questions in the interview guides were structured to unpack issues of access and usage of digital cancer care and included probes relating to the roles of various actors in the usage of digital care, preference for various digital care modalities, frequency of digital care use, experiences, and challenges during the usage of digital care. All focus groups were carried out using the Zoom video teleconferencing software, and sessions were recorded with permission. The audio files of the recordings were transcribed externally by CMBusiness & Transcription Services in preparation for data analysis.

Following the focus group interview, a self-reported sociodemographic survey was sent to each focus group participant to better understand the diversity of age, income, and other sociodemographic characteristics that were present in the focus groups. Participation in the sociodemographic survey was optional.

### Data Analysis

Qualitative data analysis followed Braun and Clarke’s 6-phase thematic analysis approach, as outlined in their original [17] and updated [18] methodological work on codebook approaches to thematic analysis. NVivo 11 software facilitated the organization and coding of data. The analysis began in February 2023 and concluded in May 2023. To ensure familiarity with the data, all authors read and re-read the transcripts. A reflexive stance was maintained throughout the analysis, with the research team acknowledging their positionality, including assumptions and experiences that may influence interpretation. A codebook was initially developed based on the key elements of the patient-centered care model [20]. Line-by-line inductive and deductive coding was conducted independently by 2 researchers (PW and AS), with additional codes iteratively developed and refined during group discussions. Disagreements in coding or categorization were resolved through collaborative reflection and consensus, ensuring confirmability of the coding process. The final coding framework was applied to all transcripts, and excerpts were attributed to relevant codes. Detailed descriptions of each code were developed to deepen understanding of participants lived and living experiences. Themes and subthemes were generated through an iterative process of synthesis and review, prioritizing coherence, internal consistency, and nuanced representation of the data.

For methodological rigor and trustworthiness of our findings, we addressed key dimensions of qualitative research. *Credibility* was ensured through investigator triangulation, as multiple team members were involved in coding and the analysis process, with group discussions validating the consistency and interpretation of the data. To support *transferability*, we provide rich, thick descriptions of participants' experiences and contextual details, enabling readers to assess the applicability of our findings to other settings. *Dependability* was established by systematically documenting each stage of the coding and analysis process, including decisions made during data synthesis, to ensure transparency and reproducibility. Finally, *confirmability* was enhanced through reflexivity, where the research team acknowledged and reflected on their positionality and potential influences on the data interpretation, alongside a collaborative approach to consensus-building during coding and theme development. These steps reinforced the rigor of our analysis and ensured a nuanced, trustworthy representation of the lived and living experiences shared by participants.

### Ethical Considerations

Institutional review board approval was provided for this research by the Toronto Women’s College Hospital Research Ethics Board (reference 2022-0111-E). The research assistant sent a detailed informed consent form by email to all potential research participants. The researchers and research assistant responded to questions from potential participants by email or by phone conversations before they signed and returned their consent forms prior to the focus group interviews. Privacy and confidentiality of research participants were ensured by removing all identification information during data transcription and analysis; nonidentificatory codes were assigned to focus group interviews that were used in this manuscript. Focus group participants were provided the option to put on or off their camera during the conversation, and we reiterated the need to keep information shared during the conversation confidential at the onset of the focus group. All patients and caregivers who participated in the focus groups were given compensation of CAD $50 (US $35.16) for their time; health care providers were not provided any compensation for participating in the research. Participants had the right to withdraw from the study at any time, without any reasons.

## Results

### Sociodemographic Results

Of the 55 sociodemographic surveys that were sent to research participants, 24 were completed by patients and caregivers (Table 2). No health care providers completed the survey. All research participants who responded to the survey and whom we identified during the focus groups self-identified as Black people.

About two-thirds of patients and caregivers who participated in the focus group identified as male, and the same proportion of participants were between 50 and 64 years old. Most participants (20/21) had college-level education or higher. More than half of the participants had part-time jobs. There was great diversity in the household income level of participants, with more than half of them earning less than CAD $60,000 (CAD $1=US $0.7) per year.

**Table 2:** Sociodemographic profile of participants.

Characteristics			Number of participants^a^
**Gender**			
		Male	16
		Female	7
**Age (years)**			
		50-64	15
		65+	7
**Highest level of education**			
		Primary or middle school	1
		Undergraduate degree	7
		College diploma or degree or certificate	6
		Master’s degree	7
**Employment**			
	Full-time		6
	Part-time		11
	Self-employed		1
	Casual		1
**Household income (CAD $^b^)**			
	0-29,999		6
	30,000-59,999		4
	60,000-89,999		3
	90,000-119,999		4
	120,000-150,000		2

^a^Numbers do not always add up to 24 because of incomplete surveys or missing data. ^b^CAD$1=US $0.70.

### Thematic Results

The experiences of patients, caregivers, and health care providers on the use of digital cancer care for older Black adults were similar across the 4 patient or caregiver groups and the 2 provider groups. In total, 5 main themes emerged from this qualitative dataset: (1) barriers to access and participation in digital care services, (2) shifting caregiver dynamics, (3) autonomy of choice and choosing based on the purpose of care, (4) digital accessibility, and (5) effective digital communication.

#### Barriers to Access and Participation in Digital Care Services

Our findings reflected that older Black patients with cancer are not a homogenous group with respect to their lived experience of digital care. They are a heterogenous group composed of individuals with unique needs and lived experiences. Additionally, individuals within this group will have experienced systems of oppression to various degrees, which has shaped the way they experienced digital cancer care. Our data show 4 main challenges that were experienced by older Black patients:

(1) *Multimorbidity* seemed to be the most salient challenge that influenced the capacity of older Black patients with cancer to participate in virtual encounters. Across the 6 focus groups, study respondents often talked about 2 main features of multimorbidity that limited the capacity of patients to effectively engage in virtual encounters. First, the severity of the patient’s cancer, marked by clinical symptoms such as intense chronic pain, loss of autonomy in activities of daily living, and persistent mental fatigue, meant that older Black patients were not always capable of fully participating in virtual encounters. Second, another aspect of multimorbidity was related to the adverse effects of chemotherapy, as explained by a caregiver.

I think sometimes we have to consider the patient’s state of mind, maybe how long the person just finished taking a medication; the amount of time before you could engage them in a virtual meeting […] you have to look at the possibility of the patient being stable before you could engage into any important communication so that you might not end up getting misinformed by the patient just because of the reactions of medications.

FG 1—Caregiver

Several respondents pointed out that chemotherapy is exhausting for most patients with cancer. Older Black patients lost appetite, had nausea and vomiting as well as fatigue after rounds of chemotherapy. These patients could not effectively participate in virtual meetings without assistance.

(2) The *geographic location* of patients emerged as another issue of concern for digital cancer care as explained by a patient:

And I will say maybe most times people in the rural areas tend not to have a good stable communication network system.

FG 4—Patient

Participants highlighted issues related to internet connection in rural and remote areas. Internet was often unreliable—they may spend days without any connections so they cannot join the virtual meeting; unstable—they may have an intermittent internet connection, so they are often disconnected from the virtual meeting; or low bandwidth—the poor quality of internet meant the connection is very slow.

(3) *Socially isolated* older Black patients were reported as having difficulties in accessing digital care. One respondent talked about a community-based initiative called the African Cancer Support Group that was launched to support socially isolated individuals of African and Caribbean descent in Alberta.

I say that because we have these groups called the African Cancer Support Group to help the friends of Africans or Caribbean and Black Canadians going through cancer. […] We’re able to provide some patients with computers.

FG 4—Caregiver

This support group identified lack of access to digital technologies such as computers, smartphones, and tablets as an obstacle to digital l care for socially isolated Black patients. One way in which they supported this disadvantaged group was by providing computers to enhance access to digital care.

(4) *Linguistic barriers* emerged as a unique challenge to digital care, as explained by a health care provider:

Language barrier is kind of something that is digging deep to the Blacks because we have different kinds of languages.

FG 5—Health care provider

Our study participants pointed out that some older Black patients with cancer were not fluent in English or French, the official languages of Canada. Health care providers were also concerned that Black seniors seemed to be more proficient in traditional African or Caribbean languages such as Swahili or Patois. These languages are not easily understood by health care professionals, and it was difficult to find trained interpreters that could facilitate communication with these patients.

#### Shifting Caregiver Dynamics

Across the 6 focus groups, there was increasing recognition of the central role of caregivers in enabling and supporting digital cancer care for older Black patients. Respondents pointed out 4 main aspects of the shifting roles of caregivers as a dynamic process:

(1) Caregivers were critical in supporting patients with limited digital literacy before, during, and after the virtual encounter:

But one of the things that was a concern for me was because my mom is elderly and she’s not really tech savvy, so I had to take charge of all portals and mail and all of that.

FG 2—Caregiver

(2) Caregivers were more involved in direct caregiving to their patient—a role that was traditionally reserved for health care providers. While family caregivers with no prior experience or health care training seemed to be less comfortable in providing direct care, paid caregivers seemed to be more comfortable in providing direct care.

(3) As caregivers were more involved in supporting digital care and providing direct care to Black seniors, there were privacy concerns related to the roles of caregivers. Specifically, some autonomous patients preferred privacy during their virtual meetings:

Like I said earlier, he has prostate cancer, and I think most times there are moments he wants to say things that are personal. So, at the moment, I like to understand his privacy and I’ll give him that privacy.

FG 4—Caregiver

Other patients with cognitive disorders who needed support from caregivers were not concerned about privacy:

So, I think in terms of privacy, because already my patient has a little bit of memory loss, so I think without me being there, I think most things would be left untold.

FG 4—Caregiver

And family caregivers, like a spouse supporting the partner in digital care, did not consider privacy an issue

I’m not worried about privacy, it’s my husband. If the patient is the wife, what kind of privacy? And mostly when you are filling the form, you put all the information they’re on the form, so they already know everything about you.

FG 4—Caregiver

(4) Some older Black patients expressed anxieties about their children who became caregivers. Specifically, the emerging role of caregivers gave rise to a shift in power or influence where caregivers are more involved in decision-making. Some older Black patients were confronted with their children having more influence in their health care—a role that children do not traditionally hold in these cultures and societies.

#### Autonomy of Choice and Choosing Based on the Purpose of Care

Focus group participants often positioned digital care dualities and in-person care as alternative and complementary means of delivering care for older Black patients with cancer.

(1) By using digital care, patients particularly appreciated the convenience and comfort of receiving care in their homes, as explained by a patient:

You can stay at the comfort of your home. And when you have a virtual conversation with your specialist, you feel this level of comfort. And you can tell your specialist how you’re actually feeling.

FG 4—Patient

Three other benefits of digital care for patients that emerged from our data included easy access to their specialist, feeling safe by not being exposed to the general population during the COVID-19 pandemic, and older Black adults felt more empowered and confident to participate in their care.

(2) Caregivers and health care providers often talked about and greatly appreciated the flexibility of digital care in adjusting their work schedules.

(3) Despite these benefits, our respondents recognized that digital care was not always realistic or possible in the continuum of cancer care. They explicitly pointed out areas of their cancer care where digital care was impractical. One patient pointed out the need for a physician examination:

I’ll say that I prefer in person because while I’m communicating in person, there are some physical examinations and some things I may want to explain myself, which I wouldn’t be able to do virtually so I prefer in person.

FG 1—Patient

(4) Some focus group members argued for more hybrid models of care delivery that integrated digital care and other care modalities like home care and ambulatory care.

#### Digital Accessibility

Study participants recognized that an important aspect of digital care meant that they had to frequently use information and communication technologies for their routine care needs. While most of our study participants were relatively comfortable with technology, certain areas of concern emerged from the interviews.

Digital literacy was identified as a problem for all participant groups—patients, caregivers, and health care providers. Older Black adults with limited digital literacy had difficulties in understanding and using computers, tablets, or applications. They needed support from caregivers and health care professionals. Interestingly, some caregivers and health care professionals also faced digital literacy issues, as explained by a health care provider:

Yes, actually, it was more time intensive, and it was more stressful because I wouldn’t say I’m that good at tech myself.

FG 6—Health care provider

The limited capacities of participants to use digital technologies could be frustrating in preparing and operating virtual encounters.

Some caregivers and health care providers mentioned that they had to invest time and efforts to improve their digital literacy skills and competencies in order to be ready to effectively support their patients.

During the pandemic, I had to learn a lot of technology skills and at the time I was involved with a virtual meeting with my patient, helping him set up virtual care with his GP and with his oncologist and every kind of health professionals involved with my patient.

FG 2—Caregiver

Certain health care organizations offered training resources to help health care providers to improve tech skills.

Finally, we noticed that older Black patients often said that they could not easily build their digital literacy skills due to their age and the burden of cancer on their daily lives. This means that the older Black patients were more reliant on their caregivers for their digital care needs. In this situation, patients seemed to value or prefer caregivers that are compassionate and patient in the way they provided support and assistance during virtual encounters:

I think for me, say one of the important things that I benefited from my caregiver, she's very patient with me. That is one thing I benefit from her. Because I think at the time, it took me almost a month to be able to navigate using virtual care. She was there for me, and her patience was something that ... I mean, I can’t comprehend on the time, because she took the time to put me through, stage by stage, for me to understand what I was doing, and how to do it.

FG 3—Patient

#### Effective Digital Communication

As focus group participants frequently talked about the importance of digital technologies as a medium for good communication and information exchange with their health care team, there were some concerns about confidentiality and privacy. Specifically, we noticed an aspect of privacy where older Black patients were reluctant to share their health information with their children, as explained by a patient:

Yeah, when I had my kids around there were times when I was uncomfortable because there were some things I couldn’t answer out loud, so I just did mute it when the question was asked and sometimes I would just tell my doctor, not now (inaudible) when we have a physical appointment or something, or maybe later when it’s just me and my wife. If it’s just my wife, I can say anything I want to say, but when my kids are around I have to limit what I say because I don’t want them having this idea that something terrible is going to happen to dad and all that.

FG 2—Patient

These older Black patients seemed to be less concerned about sharing information with their spouses.

Our focus group participants also identified other areas where digital technologies could enhance communication, such as (1) having transcripts of conversations after the virtual encounter, (2) live translations for people that speak different languages, or (3) including or using features of technology to improve care for people with visual or hearing impairments:

And when it comes to hearing impairment, I think there’s something you use in your ear that could help improve your hearing aid. So, when you’re looking at things, you have to look at these tools equally so that you don’t know whether you’re dealing with a patient that has a hearing problem or an eye problem.

FG 2—Caregiver

A summary of barriers and facilitators to digital cancer care based on interview findings is shown in Textbox 2.

**Barriers and facilitators to digital cancer care for older Black adults.**

**Barriers to digital cancer care**

- Limited engagement in virtual encounters of patients fatigued by cancer and chemotherapy or radiotherapy
- Difficulties connecting to virtual encounters due to limited and unstable internet connections in rural and remote areas
- Socially isolated older Black patients had limited access to digital technologies
- Difficult communication between patients and providers during virtual encounters due to linguistic barriers in traditional African or Caribbean languages
- Privacy concerns when caregivers supported competent patients during virtual encounters
- Difficulties in using digital care technologies due to limited digital literacy of patients, caregivers and providers
- Concerns of shifting power dynamics when children support their parents for digital cancer care
- Digital care was impractical in emergency situations

**Facilitators to digital cancer care**

- Community-based cancer support groups provided computers to socially isolated older Black patients
- Caregivers supported patients with limited digital literacy before, during, and after the virtual encounter
- Patients felt comfortable receiving digital cancer care in their homes
- Digital cancer care offered great flexibility to the work schedules of caregivers and health care providers
- Building digital literacy skills of caregivers and providers to provide better support to older Black patients
- Key features of digital technologies, such as visual and hearing aids or the capacity to have transcripts of virtual meetings, motivated actors to uptake digital cancer care

## Discussion

### Principal Findings

Inequities in cancer outcomes can be linked to higher cancer risk, delayed diagnosis, and unequal opportunities to access timely treatment. This is more significant for certain population groups that experience marginalized social conditions that are shaped by stigma, discrimination, and intergenerational trauma [15,31]. Inequities in cancer care can be further exacerbated at the intersection of social identities with other marginalizing conditions such as unequal access to the social determinants of health, resulting in health inequities that are structured and reinforced by the health care system [23]. One example of this can be the rapid shift of cancer care services from traditional in-person care towards digital cancer care models that arose in response to social distancing measures during the COVID-19 pandemic [32,33]. The implications of this shift—and the resulting impact in terms of access and optimal digital cancer care for populations that experience marginalizing social conditions—are still unknown. In our study, we sought to unpack these issues so that we could inform the redesign of digital cancer care to meet the needs and priorities of underresourced communities—thereby enabling better digital cancer care for everyone.

Prior systematic reviews reveal increasing evidence of the effectiveness of digital cancer care, which is comparable to in-person care, in counseling, and supportive medical care for people with cancer [2] and the increasing adherence of vulnerable populations, including older people and Black populations, to digital care technologies [9,34]. People living with cancer and health care providers were grateful for the convenience of videoconferencing because of reduced travel time to clinics and less travel expenses [2]. There was increasing recognition of digital health literacy challenges for older adults with investments in training older adults on the use of digital technologies and adapting these technologies to the unique needs of this population [2,9,34]. Our findings concur with previous studies on the barriers and facilitators of digital cancer care. Specifically, we identified 8 barriers to an optimal digital cancer encounter, such as linguistic barriers and limited digital literacy, and 6 facilitators that can enhance the digital cancer care experiences of older Black adults, such as community-based cancer support groups and flexible work schedules. Efforts to improve digital cancer care will require a multipronged approach that targets these specific barriers to digital care while creating system-wide policies that encourage these facilitators and influence the distribution of social determinants of health. For instance, policy interventions may be required to improve the stability of internet connections in rural and remote areas, while interpretation resource guides may help health care providers to know what translation and interpretation resources are available to them at the local or provincial and national level to overcome linguistic barriers. While these multipronged interventions will improve the digital cancer care experience for older Black adults, they are also equity-promoting for other underresourced communities and can enhance care for all.

We elaborate on 2 key findings of our study. First, our findings revealed an increasing recognition of the critical role of caregivers in supporting older Black adults in accessing and using digital cancer care. Older Black adults who are fatigued due to cancer multimorbidity, who had limited digital literacy, or who had cognitive disorders due to cancer relied on the support of caregivers before, during, and after virtual encounters. However, we also noticed that autonomous patients had privacy concerns when supported by caregivers for their digital cancer care needs. This implies that there is an urgent need for guidance at the policy and organizational level on the roles and scope of practice of caregivers in supporting digital cancer care that is tailored to the unique needs of patients with cancer.

Second, previous studies suggest that the cultural background of racialized patients influences their views on symptoms, diagnosis, and treatments that may hinder access to cancer care [31]. Our findings unraveled concerns of older Black patients with cancer who were confronted with their children having more influence in their health care during virtual encounters and who were reluctant to share their health information with their children. These dynamics between older Black patients with cancer and their children have to be taken into consideration during digital cancer care. Framing virtual cancer care in the context of a person’s cultural understanding of health requires the health care provider to have an awareness of the community that they serve [31]. This implies that efforts to develop culturally sensitive guides to digital care may be an important step to supporting health care providers to provide culturally appropriate digital cancer care to older Black patients.

### Strengths and Limitations

This study has several strengths. The use of the Synergies of Oppression framework [19] provided a robust lens to examine how intersecting systems of oppression influence access to digital cancer care for older Black patients. Including participants from 10 Canadian provinces enhanced the transferability of findings by reflecting diverse regional contexts. The focus on an understudied population—older Black adults—alongside perspectives from caregivers and health care providers offered a multistakeholder view that highlights barriers, facilitators, and opportunities for improving digital care systems. Applying Braun and Clarke’s thematic analysis [17,18] ensured a systematic and reflexive analysis, while the findings hold strong policy and practice relevance for advancing equitable, patient-centered care. Reflexivity throughout the research process further strengthened analytical transparency.

However, the study also has limitations. Conducting focus groups virtually may have introduced selection bias, as individuals with limited digital access were unable to participate. While race concordance between facilitators and participants likely fostered trust, discordance in gender, class, and affiliations may have influenced participation dynamics, and the presence of patient-caregiver dyads in the sessions may have constrained the expression of dissenting views. Additionally, regional differences across provinces were not analyzed, and findings may not reflect the full diversity within Black communities given variations in intersecting identities such as gender, class, and migration status.

Data limitations included the lack of completed socio-demographic surveys from health care providers, which restricted exploration of social factors shaping their experiences. Member checking was not conducted, though reflexivity mitigated this by ensuring transparency and neutrality. Lastly, while focus groups facilitated shared insights, they may have limited the discussion of deeply personal topics. Future research using individual interviews and exploring regional and nonusers’ experiences would provide further depth.

Despite these limitations, this study provides timely, actionable evidence to enhance digital cancer care for older Black adults. It highlights key barriers and facilitators, offers multistakeholder insights, and lays the groundwork for equitable improvements in digital health care delivery.

### Conclusions

Older Black patients face multiple barriers, such as linguistic, privacy concerns, and limited digital literacy, to accessing and using digital cancer care. A multipronged approach that focuses on addressing barriers, encouraging facilitators, and creating culturally sensitive guides to digital care can form the basis of health system efforts to improve access to digital cancer care. A redesign of digital cancer care programs, tailored to the needs of marginalized social groups like older Black patients, can enhance the digital care experience for all population groups. Public policies and organizational practices that address issues like availability of internet in remote areas, resources to support linguistic barriers, or culturally sensitive training are important in responding to the complexity of access to digital cancer care.

## Appendix

Multimedia Appendix 1 Combined caregiver and patient discussion guide.

Multimedia Appendix 2 Virtual cancer care provider discussion guide.

## Abbreviations

CCS: Canadian Cancer Society

## Data Availability

The datasets generated and analyzed during this study are not publicly available as they include confidential data, but are available upon request from the corresponding author, including the need of Women’s College Hospital ethical vetting.
