# Supplementary material for: Improving Digital Cancer Care for Older Black Adults: Qualitative Study
Source: J Med Internet Res. 2025 Feb 19;27:e63324. doi: 10.2196/63324 (PMC11888062; doi:10.2196/63324)
Supplement: Multimedia Appendix 1 [file jmir_v27i1e63324_app1.docx]

**Combined Caregiver & Patient Discussion Guide (Appendices B &C)**

**Preamble**

The purpose of this study is to inform the co-design of strategies to optimize virtual cancer care for older adults in the Black community, their caregivers, and healthcare providers. We’ve invited you to this one-time, two hour-long focus group session to learn more about your experiences with virtual cancer care, including some of the challenges you may have experienced, your opinion on the benefits of virtual care, and what changes you would like to see introduced to virtual cancer care. We hope to get your insights about how you think virtual cancer care programs can be designed to better support health equity. The information collected from you will be used to inform future design activities led by the Canadian Cancer Society regarding the development of best practices for equitable virtual cancer care.

Before we begin:

- Does anyone have any questions about the study?
- Does anyone have questions regarding their participation in this focus group?

**Key points for consent form**: We want to assure everyone here that you can refuse to answer any questions you don’t want to, and you can end your participation in the focus groups anytime you like. We will take all necessary precautions to protect your confidentiality, but we cannot guarantee that other members of the focus group will respect your privacy or keep the discussions of the group confidential. We implore everyone here to use their best judgement in being discrete about the nature of these conversations and respecting others’ privacy.

- Are there any other aspects of the consent form that anyone would like me to review?
- Could you all confirm that I have your permission to audio record this session? [Wait for confirmation, then start audio recording]
- Could you also confirm that you’re providing me with your verbal consent to participate in this study? [Wait for confirmation before starting the session]

**Opening questions (EVERYONE)**

- Share an aspect of your life experience that has brought you here today.
- Why did you decide to join our focus group today?

**Background Information (CAREGIVERS)**

- How long have you been a caregiver?
- Who are you a caregiver to?
- Do you live with the care recipient?
- are you involved in their virtual appointments? If so, how? (e.g., travel to care recipients’ home, care recipient travels to caregiver’s home, 3-way interaction with providers)

**Role Clarity (CAREGIVERS)**

- Are you involved in all the care recipients’ cancer-related appointments? What virtual appointments did you participate in? What was the purpose of these appointments?
  - Prevention discussions
  - Early detection and diagnosis-related appointments
  - Treatment discussions
  - Recovery and follow-up appointments
  - Counselling or palliative care
  - End of life care
- What is the extent of your involvement in these virtual interactions? (e.g., only to provide device/setup support, present and actively involved in the whole appointment, advocacy support, translation support, etc)
- Is your role as a caregiver clear to you? If no, what are you unsure about? (e.g., access to personal information, involvement in decisions, etc)

**Caregiver and family involvement (PATIENTS)**

- Do you have a caregiver? Does anyone in your family support you during any aspect of your virtual appointments? If yes:
- How have you benefited from the support of your caregiver/family member during virtual appointments?
- In what capacity is your caregiver involved in your virtual appointments?
  - Provides access to devices
  - Provides set-up support
  - Help with translation/explaining complex medical jargon
  - Supports decision-making
- Does the involvement of your caregiver/family member present any challenges? (e.g., lack of privacy to discuss sensitive information, caregiver opinion taken at greater value)
- In what ways is caregiver/family involvement different between in-person care and virtual care?

**(Background Information)_ Clinical purpose of virtual interaction (PATIENTS)**

- What did you use virtual care for?
  - Prevention discussions
  - Early detection and diagnosis-related appointments
  - Treatment discussions
  - Recovery and follow-up appointments
  - Counselling or palliative care
  - End of life care

**Impression of virtual cancer care (EVERYONE)**

- What words or feelings come to mind when you think about virtual care?
  - How did you form this impression?
- What was your first reaction to virtual care?
  - How did your opinion of virtual care change over time?

**Preferences for care modality (EVERYONE)**

- Which virtual medium would you prefer to communicate in with the recipient’s health care providers? (e.g., by phone, video, text messaging, email, etc).
  - Would this preference change depending on the nature of the visit? If so, how?
- If the choice was up to you, what factors would influence whether you and the care recipient have an in-person appointment or a virtual appointment?
- Which care modality would you prefer for the following purposes?
  - Prevention discussions
  - Early detection and diagnosis-related appointments
  - Treatment discussions
  - Recovery and follow-up appointments
  - Counselling or palliative care
  - End of life care
- Do you know what the care recipients’ preferences are? Are they different from yours?

**Frequency of virtual care use (EVERYONE)**

- How often did you participate in virtual interactions with your care recipient and their providers over the last 2 years?
- When was the last time you had one of these virtual interactions?

**Virtual Care Experience (CAREGIVERS)**

- How has virtual care impacted your experience as a caregiver?
- How has your experience as a caregiver improved due to virtual care? What are the benefits of virtual care?
- What benefits does virtual care provide over in-person care?
- Does virtual create any issues that are not present during face-to-face encounters?

**Pros and Cons of virtual cancer care (PATIENTS)**

- What are your favorite and least favorite aspects of virtual care?
- What do you think are the strengths and weaknesses of virtual care?
- What benefits does virtual care provide over in-person care?
- Does virtual create any issues that are not present during face-to-face encounters?

**~~Caregiver and Care Recipient~~ Challenges (EVERYONE)**

- What do you think these virtual interactions are missing?
- What kinds of problems have you, as a caregiver, experienced when using virtual care?
  - Increased caregiver burden (e.g., provide access to device, troubleshoot connection issues, create accounts, etc)
  - Not sure when to step away to provide care recipient with privacy
  - Access to sensitive information (e.g., EMRs, patient portals)
- What kinds of challenges has your care recipient experienced? Have you experienced some of the same challenges? Did it become your responsibility to find solutions to these challenges?
  - Digital health literacy
    - the knowledge of how to use a specific technology for a health care purpose and the confidence to act on that knowledge to participate in a virtual appoint)
  - Access to devices (e.g., smartphones, laptops, tablets)
  - Access to Internet
  - Cost
  - Self-efficacy (confidence/comfort with using technology)
  - Privacy concerns
  - Peer support
  - Physical, cognitive, sensory limitations (e.g., visual, auditory/hearing concern)
  - Language barriers
  - Cultural relevance

(& PATIENTS CAN YOU TELL US ABOUT ANY CHALLENGES YOU EXPERINCED DURING VIRTUAL CARE

- - Digital health literacy
    - the knowledge of how to use a specific technology for a health care purpose and the confidence to act on that knowledge to participate in a virtual appoint)
  - Access to devices (e.g., smartphones, laptops, tablets)
  - Access to Internet
  - Cost
  - Self-efficacy (confidence/comfort with using technology)
  - Privacy concerns
  - Peer support
  - Physical, cognitive, sensory limitations (e.g., visual, auditory/hearing concern)
  - Language barriers
  - Cultural relevance

**Caregiver Support (CAREGIVERs)**

- - How can organizations support your experience before the appointment?
    - Staff support (e.g., downloading apps, creating accounts for the care recipient)
    - Instructional guides for caregivers
  - During the appointment? (e.g., live tech/trouble-shooting support)
  - After the appointment? (e.g., follow up information/appointment summary sent to caregiver)

**Comparison of virtual cancer care to other virtual interactions (EVERYONE)**

- Are you involved in other virtual health appointments with the care recipient that are unrelated to cancer care?
- How does your experience with virtual cancer care compare to your experience with virtual care for other clinical purposes?
- Do you use virtual/digital technologies with the care recipient for purposes outside of health care? If so, what?
  - How do these experiences compare to those of virtual health care?
  - Is there anything about these experiences you would like to see in virtual health care?

**Improvements to virtual care processes (EVERYONE)**

- If you could change one thing about virtual care, what would it be?
- What would you most like to add to or improve about virtual care? How could providers/organizations improve your virtual care experience?
  - How can organizations support your experience before the appointment?
    - Staff support (e.g., downloading apps, creating accounts)
    - Instructional guides
  - During the appointment? (e.g., live tech/trouble-shooting support)
  - After the appointment? (e.g., instructions/appointment summary sent via email)

**Closing questions (EVERYONE)**

- Is there anything else you’d like to say about your experience with virtual care?
- Is there anything we haven't touched on today that you would like us to know?
