# Supplementary material for: Improving Digital Cancer Care for Older Black Adults: Qualitative Study
Source: J Med Internet Res. 2025 Feb 19;27:e63324. doi: 10.2196/63324 (PMC11888062; doi:10.2196/63324)
Supplement: Multimedia Appendix 2 [file jmir_v27i1e63324_app2.docx]

**Appendix D – Virtual Cancer Care Provider Discussion Guide**

**Preamble**

The purpose of this study is to inform the co-design of strategies to optimize virtual cancer care for older adults in the Black community, their caregivers, and healthcare providers. We’ve invited you to this one-time, two hour-long focus group session to learn more about your experiences with virtual cancer care, including some of the challenges you may have experienced, your opinion on the benefits of virtual care, and what changes you would like to see introduced to virtual cancer care. We hope to get your insights about how you think virtual cancer care programs can be designed to better support health equity. The information collected from you will be used to inform future design activities led by the Canadian Cancer Society regarding the development of best practices for equitable virtual cancer care.

Before we begin:

- Does anyone have any questions about the study?
- Does anyone have questions regarding their participation in this focus group?

**Key points for consent form**: We want to assure everyone here that you can refuse to answer any questions you don’t want to, and you can end your participation in the focus groups anytime you like. We will take all necessary precautions to protect your confidentiality, but we cannot guarantee that other members of the focus group will respect your privacy or keep the discussions of the group confidential. We implore everyone here to use their best judgement in being discrete about the nature of these conversations and respecting others’ privacy.

- Are there any other aspects of the consent form that anyone would like me to review?
- Could you all confirm that I have your permission to audio record this session? [Wait for confirmation, then start audio recording]
- Could you also confirm that you’re providing me with your verbal consent to participate in this study? [Wait for confirmation before starting the session]

**Opening questions**

- Share an aspect of your life experience that has brought you here today.
- Why did you decide to join our focus group today?

**Community/patient overview**

- Can you tell us a little bit about the community you serve?
  - Overview of patients/clients.
  - What kinds of diversity is present?

**Impression of virtual cancer care**

- What was your first reaction to virtual care? How has your opinion on virtual care changed over time?
- What do you think are the strengths and weaknesses of virtual care?
- How comfortable/confident are you with using technology?
  - What steps can organizations take to improve your comfort with technology?
  - What steps were taken to increase your confidence in using virtual care?
- What steps did your organization take to support the seamless integration of virtual care into your practice?

**Benefits of virtual cancer care**

- What do you like about virtual care?
- How has virtual care improved your interactions with patients? With caregivers? Family members?
- In what ways has virtual care made your job easier?
- What advice would you give to providers using virtual care for the first time?
- What advice would you give to providers who want to use virtual more frequently?

**Care modality**

- What factors do you consider when choosing a type of virtual care medium (e.g., phone, video, text messaging, email, etc.) for appointments?
- What influences whether patients are given an in-person appointment or a virtual appointment?
- Were you provided with any any directive resources to support you in making these decisions?
- Were there any changes to your normal work routine/workflow? Please describe.

**Challenges experienced during virtual interactions**

- What do you think virtual care is missing?
- What kind of problems have you experienced when using virtual care?
  - Digital health literacy
  - Self-efficacy
  - Concerns about quality / health outcomes
  - Increased workload/administrative burden

**Training/staff support**

- What kinds of supports and/or training were you given to engage in virtual care?
  - Did your organization provide online training services?
  - Did your organization develop their own guidelines?
- What types of training would you like to see moving forward?

**Patient and caregiver/family considerations**

- Did your organization engage with and/or consult with community organizations and/or patients, caregivers, or family members regarding virtual care planning and delivery?
- Were special considerations made for different populations? If yes, what were these considerations?
- How were individual determinants such as language, race/ethnicity, ability, income, or age specifically considered by your organization?
- Does your organization have a way of measuring provider experience with virtual care? Patient experience?
  - Does your organization measure satisfaction with virtual care? How?
  - Does your organization measure quality of virtual care service (e.g., efficiency, impact, effectiveness)? How?
- Do you think there’s a better way of measure key performance indicators? Please elaborate.

**Improvements to virtual care processes**

- What would you most like to add to or improve about virtual care?
- What steps would you like to see from your organization to support virtual care delivery?

**Closing questions**

- Is there anything else you’d like to say about your experience with virtual care?
- Is there anything we haven't touched on today that you would like us to know?
